# Cervicofacial Cellulitis due to *Staphylococcus aureus* with Jugular Vein Thrombosis and Multiple Septic Pulmonary Embolism: A Lemierre-Like Syndrome

**DOI:** 10.1155/2022/7805523

**Published:** 2022-08-26

**Authors:** Ibrahima Niang, Latyr Junior Diouf, Papa Abdou Diop, Daouda Thioub, Alassane Sarr, Khadidiatou Ndiaye Diouf, Geraud Lera Akpo, Abdoulaye Dione Diop, Sokhna Ba

**Affiliations:** ^1^Radiology Department, Fann University Hospital Center, Dakar, Senegal; ^2^Infectious Diseases Department, Fann University Hospital Center, Dakar, Senegal

## Abstract

This is the case of a 28-year-old male patient with no particular pathological history who presented with an inflammatory swelling of the right cheek with pus in an infectious context. Cervicofacial CT with contrast injection allowed the diagnosis of a right cervicofacial cellulitis, associated with a jugular venous thrombosis extending to the superior vena cava. It also revealed septic pulmonary metastases in the form of multiple excavated pulmonary nodules. The analysis of the pus sample allowed the isolation of *Staphylococcus aureus* as the causative germ. This led to the diagnosis of a Lemierre-like syndrome, which is a variant of the Lemierre syndrome. Despite appropriate antibiotic treatment and anticoagulation, the patient died after 16 days of hospitalization.

## 1. Introduction

The Lemierre syndrome is an uncommon condition with a prevalence of 0.8 cases per million population [1, 2]. It corresponds to a jugular venous thrombosis during oropharyngeal infection by an anaerobic germ (classically, *Fusobacterium necrophorum*) and may be complicated by septic metastases, most often pulmonary [3, 4]. In the presence of jugular venous thrombosis in the context of a non-oropharyngeal or aerobic infection, it would be more appropriate to call it a Lemierre-like syndrome [5, 6]. Cervicofacial cellulitis is one of the most frequent head and neck infectious emergencies and also one of the most serious [7]. Moreover, *Staphylococcus aureus* is the most common identified cause of cellulitis [8].

We report the case of a 28-year-old male patient with cervicofacial cellulitis due to *Staphylococcus aureus* and complicated by right jugular vein thrombosis and multiple pulmonary septic metastases, thus corresponding to a Lemierre-like syndrome.

## 2. Case Presentation

**Figure 1:**
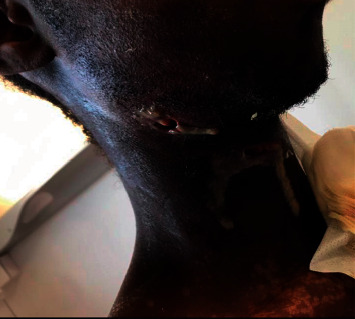
Photograph showing the right jugal swelling with fistulizations under the right maxilla and cervical leaving pus (white arrows).

This is a 28-year-old male patient who consumed alcohol and tobacco (quantity unspecified), with no reported pathological history, no intravenous drug use, or known risk of immunodepression received for a right cervicofacial inflammatory swelling with fistulization to the skin, leaking pus (Figure 1). The onset of these symptoms was about one week ago, marked by a right jugal inflammatory swelling without signs of tonsillitis. On admission, the patient was in a bad general condition with clear consciousness. He was pyretic with a temperature of 39 C, the rest of his vitals were heart rate of 130 bpm, respiratory rate of 22 breaths per minute, blood pressure of 143/98 mm·Hg, and an oxygen saturation level of 97%. He had a poor oral status and the pulmonary examination revealed a left basal fluid effusion syndrome and bilateral pulmonary condensation syndrome. The rest of the examination was unremarkable.

Laboratory examinations revealed the following results: hemoglobin 11.2 g/dl, WBC 39900/mm^3^ (neutrophils 89%), C-reactive protein 261.6 mg/l, creatinine 76.5, and fasting blood glucose 0.8 g/L.

Serologic tests for antibodies to hepatitis viruses B and C, human immunodeficiency viruses 1 and 2, were negative. The Emmel test for sickle cell was positive with an AS profile at electrophoresis.

**Figure 2:**
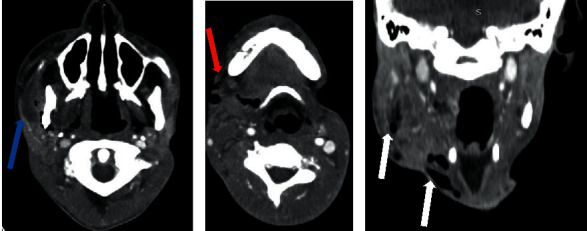
Cervical CT scan with contrast injection. (a) Axial view showing right masticatory area fluid collection (blue arrow). (b) Axial view showing fistulous path to the skin (red arrow). (c) Coronal view showing heterogeneous content of the collection with air bubbles (white arrows).

**Figure 3:**
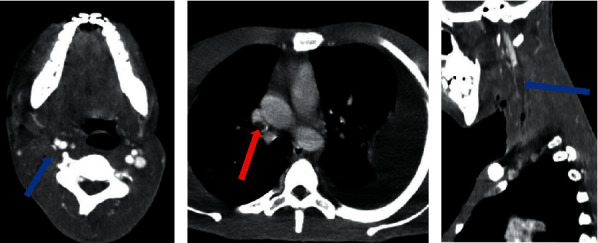
Cervicothoracic CT scan with contrast injection. (a) Axial view at cervical level showing occlusive thrombosis of the right jugular vein (blue arrow). (b) Axial view at thoracic level showing thrombosis of the right superior vena cava (red arrow). (c) Sagittal view showing the thrombosis extended along the right jugular vein to the superior vena cava (blue arrow).

**Figure 4:**
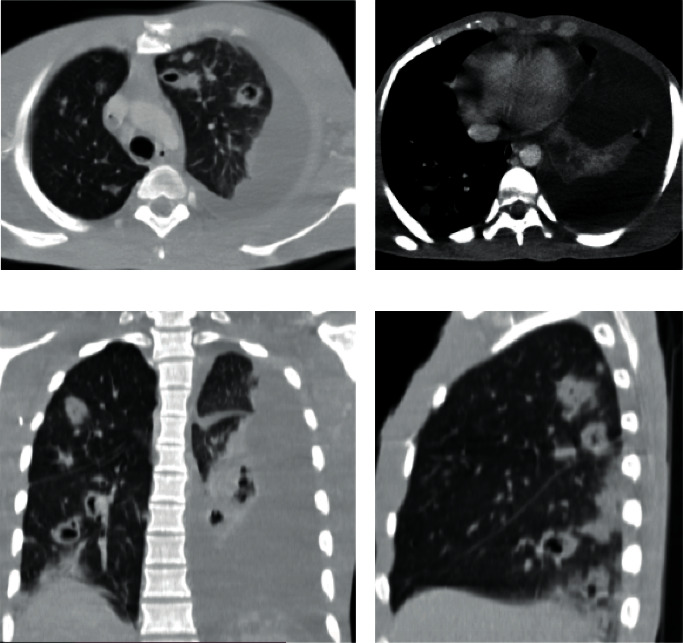
Thoracic CT scan with contrast injection. (a, c, and d) Axial, coronal, and sagittal views in the lung window showing multiple bilateral excavated nodules. (b) Axial view in mediastinal window showing left pleural fluid effusion with passive atelectasis of the lower lobe.

A cervicothoracic CT scan was performed in this context. It showed a cervicofacial cellulitis with a collection in the right masticatory region, heterogeneous with air bubbles and extended to the cervical region with a fistulous path (Figures 2(a)–2(c)). It also visualized a thrombosis of the right internal jugular vein extended to the superior vena cava (Figures 3(a) and 3(b)). At the thoracic level, the CT scan showed multiple bilateral diffuse pulmonary nodules, most of which were excavated, and a moderate amount of left pleural fluid effusion (Figures 4(a)–4(c)). The drainage of this pleural effusion brought back 350 cc of a citrine yellow liquid. There was no pulmonary embolism or other cervicothoracic lesion.

Analysis of the pus sample identified methicillin-resistant *Staphylococcus.* Blood cultures were not taken. The patient had initially received empirical intravenous antibiotic therapy with ceftriaxone (2 g/day) and metronidazole (500 mg × 3/day) and then three days later, adapted with vancomycin (500 mg × 3/day). He had also received anticoagulant treatment with rivaroxaban (15 mg × 2/day).

After 13 days in intensive care, the patient's condition had improved with apyrexia, a decrease in hyperleukocytosis to 21,000/mm^3^ and CRP to 121 mg/L, motivating his transfer out of intensive care.

At day 16 of hospitalization, the patient had an episode of massive hemoptysis with cardiorespiratory distress leading rapidly to his death. An autopsy was not authorized by his parents.

## 3. Discussion

The Lemierre's syndrome, also called necrobacillosis, is a serious complication of oropharyngeal infection, most often due to an anaerobic germ and responsible for jugular thrombosis and distant septic metastases, most often pulmonary [4]. The germ involved is most often *Fusobacterium necrophorum* [3]. Cases of jugular thrombosis in the context of an infection whose origin is not oropharyngeal and whose germ is aerobic should be called a Lemierre-like syndrome because they do not correspond to the original definition [5, 6]. First reported in 1936 by André Lemierre in a series of 20 cases, the Lemierre syndrome was very rare in the literature after 1940, to the point of being called “the forgotten disease” [2, 9]. The rarity of cases of Lemierre syndrome during this period would be due to the advent of antibiotics and their early use in ENT infections [10]. Currently, over the past 20 years, the number of reported cases of the Lemierre syndrome has been increasing [11, 12]. This may be related to an increase in cases due to antibiotic resistance or overuse of NSAIDs in ENT infections or just a greater trend to publication [4].

Among these new cases of the Lemierre syndrome, some are due to other germs other than *Fusobacterium necrophorum* and even to aerobic germs with other non-oropharyngeal infectious sites [5, 11]. Thus, not all of these new cases meet the exact definition of the Lemierre syndrome and for some authors, these cases should be reported as the Lemierre-like syndrome [6, 13].

These cases of the Lemierre-like syndrome due to *Staphylococcus aureus* are reemergent and most often found in young healthy adults [5]. This was the case in our patient in whom no medical history or immunosuppressive factors were found. In our patient, the initial infection identified was a cervicofacial cellulitis with no reported oropharyngeal or dental infection. Moreover, our patient had taken nonsteroidal anti-inflammatory drugs at the beginning of his symptomatology. This is certainly at the origin of the extension of the cellulitis, going as far as forming collections and fistulas. The use of NSAIDs is a factor that is often present and should be investigated, particularly in cellulitis caused by *Staphylococcus* [8, 14]. The common element between the Lemierre syndrome and Lemierre-like syndrome is jugular venous thrombosis in an infectious context, hence the importance of imaging (Doppler ultrasound, CT, or MRI) in their diagnosis. Thus, in case of any oropharyngeal or cervicofacial infection that does not improve, it is necessary to think of a Lemierre syndrome or Lemierre-like syndrome and to look for a jugular venous thrombosis by imaging. CT with contrast is the best modality because it allows both visualization of the jugular thrombosis and the extension of the cervicofacial infection [15]. This thrombosis could be explained by a state of hypercoagulability due to the infection and endothelial lesions due to the inflammation [16].

The other major complication frequently found in the Lemierre syndrome or Lemierre-like syndrome is septic metastasis, the main location of which is the lung [17, 18]. Hence, the importance of watching for pulmonary signs (cough, dyspnea) and not hesitating to perform a thoracic CT scan. These septic lung metastases (or septic lung emboli) present as multiple bilateral pulmonary nodules, most often excavated [10, 18]. Other less common locations of these septic metastases are splenic hepatic and articular [19]. It is important to initiate antibiotic treatment before the occurrence of these life-threatening septic metastases. This antibiotic therapy must be broad spectrum from the outset and then adapted to the germ identified. In our patient, the germ involved was *Staphylococcus aureus*. This germ is found with great frequency in skin or soft tissue infections, as in our patient's case [7].

In addition to antibiotic therapy, our patient had received anticoagulant treatment. However, this anticoagulant treatment is controversial because for some it would increase the risk of migration of septic emboli and for others it would improve the penetration of the antibiotic [10, 16]. The prognosis of Lemierre's syndrome was severe before the advent of antibiotics, and of the 20 patients initially reported by André Lemierre, 18 died. While currently the mortality rate is estimated to be 4 to 18% [20]. The unfavorable prognosis in our patient could be explained by a delay in diagnosis due to the use of NSAIDs but also by the multiple pulmonary septic emboli.

## 4. Conclusion

In conclusion, we would say that Lemierre's syndrome should be distinguished from Lemierre-like syndromes whose common point is the jugular venous thrombosis in an infectious context. In this case, *Staphylococcus aureus* is to be considered as a germ, especially for cervicofacial infections of the skin and soft parts (cellulitis and necrotizing fasciitis). Pulmonary septic emboli are to be feared and an adapted and early antibiotic treatment is necessary to avoid a fatal outcome.

## Data Availability

No data were used to support this study.
